# Male breast cancer in Arab populations: a systematic review of molecular, genetic, and clinicopathologic characteristics

**DOI:** 10.1186/s43046-026-00408-0

**Published:** 2026-09-21

**Authors:** Shaden Abunasser, Raghad Al-Taweel, Shahrazad Elnatsheh, Areej Baraka, Muna Al-Ismail, Waad Rashid Aldosari, Baraah A. Alquran, Atiyeh M. Abdallah, Amal Al-Haidose

**Affiliations:** 1 Qatar University, Doha, Qatar; 2 Ministry of Interior, Doha, Qatar; 3 Jordan University of Science and Technology, Irbid, Jordan

**Keywords:** Male breast cancer, Arab region, Molecular subtypes, Hormone receptor status, Genetic alterations, BRCA2

## Abstract

**Background:**

Male breast cancer (MBC) accounts for less than 1% of all breast cancer cases, yet its incidence is increasing worldwide. Research on MBC remains limited, particularly in Arab populations, where molecular and genetic characteristics are poorly characterized. Improved understanding of MBC biology is essential to support region-specific, biology-driven management strategies.

**Methods:**

This systematic review was registered in PROSPERO (CRD42024607019) and conducted according to PRISMA guidelines. PubMed, Embase, and Scopus were searched from inception to 28 December 2025. Eligible studies reported MBC cases from Arab countries and included data on molecular subtypes, receptor status, genetic alterations, and/or clinicopathologic characteristics. Data were extracted independently by four reviewers. Risk of bias was assessed using the Joanna Briggs Institute (JBI) critical appraisal tool, and findings were synthesized narratively with harmonization of tumor grade and stage where applicable.

**Results:**

From 9,337 records, 54 studies comprising 1,534 MBC patients met the inclusion criteria. Invasive ductal carcinoma was the predominant histological subtype (92.6%). Among patients with reported intrinsic subtypes (*n* = 335), luminal A (45.7%) and luminal B (40.9%) accounted for over 86% of cases, whereas HER2-positive (4.8%) and triple-negative (8.4%) tumors were uncommon and triple-positive disease was rare (0.3%). Receptor status was reported for 1,031 patients, with high ER (71.5%) and PR (71.6%) positivity and low HER2 positivity (12.6%), alongside substantial heterogeneity in testing and reporting. Genetic data were available for 59 patients, most frequently identifying pathogenic truncating *BRCA2* variants, although BRCA-negative cases were also reported. Surgery was the most reported treatment modality (*n* = 856), predominantly mastectomy-based, while endocrine therapy was frequently used and HER2-targeted therapy was infrequently reported.

**Conclusion:**

MBC in Arab populations is predominantly hormone receptor–driven, characterized by luminal subtypes, high ER/PR positivity, and low HER2 expression. Genetic evidence, though limited, implicates *BRCA2* as the most frequently reported susceptibility gene. These findings highlight the need for standardized molecular reporting and broader access to receptor profiling and genetic testing to support personalized, region-specific MBC management.

**Supplementary Information:**

The online version contains supplementary material available at https://doi.org/10.1186/s43046-026-00408-0.

## Introduction

Male breast cancer (MBC) is a rare disease, accounting for less than 1% of all breast cancer cases, yet its incidence has been steadily increasing worldwide [1]. Similar to female breast cancer (FBC), MBC risk increases with age; however, men are typically diagnosed approximately a decade later, with a peak incidence around 70 years of age [2]. Despite this rising incidence, research on MBC remains limited, as most clinical research and therapeutic advances have historically focused on FBC. Consequently, diagnostic and treatment strategies for MBC are largely extrapolated from FBC guidelines, which may not fully capture the biological and clinical characteristics unique to men with breast cancer [3]. Delayed diagnosis is common in MBC, partly due to lower awareness and less frequent use of diagnostic imaging, often leading to more advanced disease at presentation [3].

Breast cancer is classically categorized into four key molecular subtypes, luminal A, luminal B, HER2-enriched, and triple-negative, based on the expression of surface receptors including the estrogen receptor (ER), progesterone receptor (PR), and human epidermal growth factor receptor 2 (HER2), each with important prognostic and therapeutic implications [4]. While these subtype frameworks are routinely applied to MBC, accumulating evidence suggests that MBC displays distinct molecular and clinicopathological features, including a predominance of hormone receptor-positive disease and lower rates of HER2 overexpression, which may influence treatment responses and outcomes [5–7]. Therefore, relying solely on FBC standards of care may overlook clinically meaningful differences in MBC biology.

Genetic predispositions, such as *BRCA1* and *BRCA2* mutations, appear to play a crucial role in MBC risk, with prevalence and impact varying across populations due to regional genetic, environmental, and lifestyle factors [1, 7]. For example, MBC is usually the invasive ductal adenocarcinoma histopathological subtype, and they are more often ER and PR positive and less likely to overexpress HER2 [8, 9]. Despite these findings, there have been few genetic studies on MBC, especially in the Arab region, leaving possible regional variations underexplored. It is essential to understand and investigate these genetic profiles and interactions to develop effective, region-specific MBC treatment and management strategies.

Although research on MBC has expanded in Western countries, data from the Arab region remain limited and fragmented, despite evidence of regional variability in incidence and tumor characteristics [3]. Currently, no established standard-of-care exists for MBC, highlighting the critical need for focused research, as improved characterization of molecular subtypes and genetic profiles may enable more personalized, biology-driven, and region-specific therapeutic strategies [10].

A previous systematic review of the epidemiological, clinicopathological, and treatment-related aspects of MBC in Arab countries based on studies published through February 2022 did not specifically focus on molecular subtype classification or genetic alterations, nor did it capture more recent literature [11]. Therefore, here we provide an updated and focused synthesis of molecular subtypes and genetic variations in MBC across Arab populations using a comprehensive multi-database search strategy and extending the search period through to December 2025. By systematically characterizing subtype distributions and reported genetic alterations in this underserved region, this review aims to improve biological understanding, inform region-specific therapeutic strategies, and highlight critical gaps for future research.

## Materials and methods

### Search strategy and objectives

This systematic review protocol was registered with the International Prospective Register of Systematic Reviews (PROSPERO) under the registration ID CRD42024607019. Preferred reporting items for systematic review and meta-analysis (PRISMA) guidelines were followed [12] (Supplementary Table S1). The primary objective of this systematic review was to characterize the molecular subtypes and genetic variations of MBC within the Arab population, where the Arab region was defined according to the United Nations classification of the League of Arab States and includes the 22 member states of Bahrain, Iraq, Egypt, Jordan, Kuwait, Lebanon, Qatar, the state of Palestine, Saudi Arabia, the Syrian Arab Republic, Oman, United Arab Emirates, Yemen, Algeria, Tunisia, Morocco, Libya, the Comoros, Djibouti, Mauritania, Somalia, and Sudan .

The PubMed, Embase, and Scopus databases were searched from database inception to 28 December 2025, using MBC-related keywords and controlled vocabulary terms where applicable. Keywords included *“male breast cancer”*, *“breast cancer in men”*, *“male breast carcinoma”*, and *“breast tumors in men”*. MeSH vocabulary terms were used when available (e.g., *Breast Neoplasms*,* Male* in PubMed) (Supplementary Table S2). Review articles were excluded by specifying publication type and title within the search strategy. No language restrictions were applied during the search or study selection process. Articles published in languages other than English were translated where necessary for eligibility assessment and data extraction. Grey literature sources (e.g., conference proceedings, dissertations, and trial registries) and preprint servers were not searched. Country names were not explicitly included in the search strategy to avoid omission of relevant studies in which geographic origin was not specified in titles or abstracts. Geographic eligibility was therefore determined during full-text screening. Titles and abstracts were screened for relevance, followed by full-text assessment of eligible studies for inclusion in the final analysis.

### Study selection

**Fig. 1:**
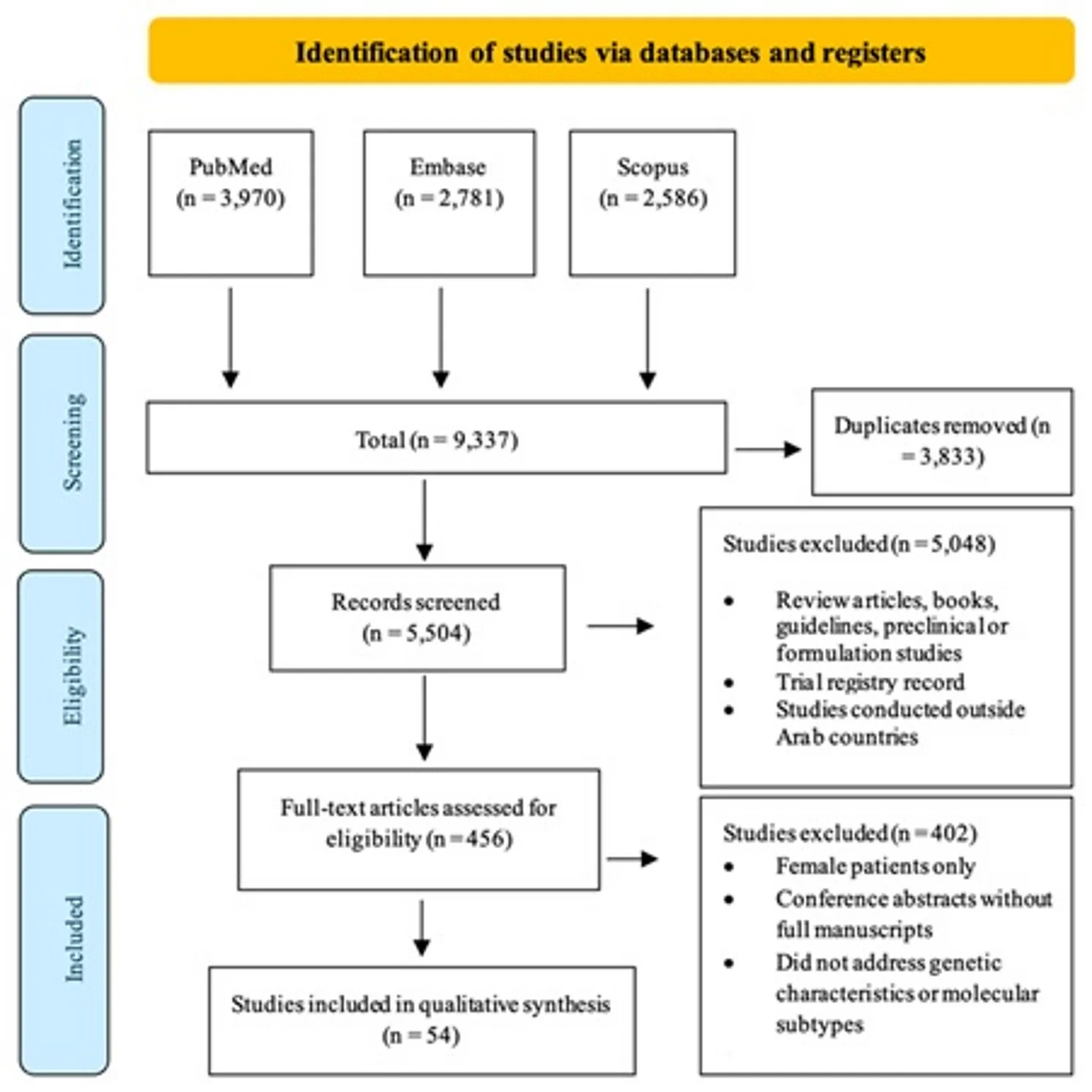
PRISMA flow chart of the screening and selection process

The total number of articles retrieved from each database was recorded. Eligible studies were selected according to the PICOS criteria: (1) peer-reviewed journal articles; (2) MBC patients from Arab countries as defined by the UN; and (3) reporting molecular subtypes, genetic alterations, or clinicopathological characteristics of MBC. Some papers were also excluded after full review because they lacked specific information relevant to the focus of this study. Following duplicate removal, titles and abstracts were screened for relevance, and full texts of potentially eligible articles were subsequently assessed. Additionally, papers without available abstracts and with no accessible full text were also excluded from the study. All screening and selection steps were independently conducted by four reviewers, with discrepancies resolved through consultation with a senior researcher. Reference list of included studies was not manually screened for additional eligible studies, as study identification was restricted to the predefined electronic database search strategy to maximize reproducibility. As consensus was reached for all included records, inter-rater reliability statistics were not calculated. The study selection process is illustrated in Fig. 1.

### Data collection

Four researchers independently used a specific structured approach to extract data from the included studies, and a senior researcher assessed the data extraction process. The data elements included: (1) study characteristics (first author’s name, publication year, recruitment period, study location, sample size); (2) patient characteristics (mean or median age, sex, histological subtype, tumor size, tumor stage, metastasis, and nodal status); (3) outcomes (molecular subtype, receptor status, *BRCA1/BRCA2* mutation status, treatment received, if available).

### Quality control assessment and data extraction

Two researchers assessed the methodological quality of included studies using the Joanna Briggs Institute (JBI) critical appraisal tools checklists according to study design (case reports, case series, cohort studies, and cross-sectional studies). The JBI tools are widely recognized as reliable and validated instruments for assessing methodological quality and risk of bias in clinical and observational studies [13, 14]. An overall risk of bias judgment (low, moderate, or high) was assigned for each study based on the available information. The overall distribution of risk of bias across studies was presented using a graphical representation. Discrepancies were resolved through consultation with a senior researcher.

### Data synthesis and reporting

The feasibility of a quantitative meta-analysis, including meta-analysis of proportions, was considered during the review process. However, the included studies were not considered sufficiently comparable for meaningful quantitative synthesis because of substantial clinical and methodological heterogeneity [15]. Specifically, the studies differed in study design (case reports, case series, retrospective cohort studies, and cross-sectional studies), sample size, patient populations, molecular subtype definitions, completeness of receptor and genetic reporting, and genetic testing methodologies. Furthermore, several outcomes were inconsistently reported or incompletely described, limiting the comparability of prevalence estimates across studies. Therefore, the findings were synthesized narratively and summarized descriptively. Study characteristics and outcomes were tabulated and presented in summary tables and figures to facilitate comparison of histopathological features, molecular subtypes, and genetic variations. Frequencies and proportions were calculated for categorical variables where appropriate, and all extracted data were organized and verified using Microsoft Excel.

To ensure comparability across studies, data were harmonized prior to synthesis. Tumor grade was standardized across different grading systems, including the Scarff–Bloom–Richardson (SBR) system and the Van Nuys classifications, and reclassified into unified grade categories (G1–G3) when sufficient information was available. Similarly, tumor stage was harmonized across staging frameworks, including the American Joint Committee on Cancer (AJCC) and the Union for International Cancer Control (UICC) classifications. When any outcome was reported only as a percentage, absolute counts were recalculated based on reported sample sizes. Percentages presented in summary tables were recalculated using pooled denominators to ensure consistent and standardized reporting across studies.

Intrinsic molecular subtype classifications were extracted as reported in original studies. No attempt was made to assign or reclassify tumors into intrinsic molecular subtypes using surrogate immunohistochemical criteria (e.g., ER, PR, HER2, Ki-67, or tumor grade) when these classifications were not explicitly reported by the original authors. Consequently, only studies that explicitly reported intrinsic molecular subtype classification were included in the molecular subtype analysis (Table 1).

**Table 1:** Inclusion and exclusion criteria according to the PICOS statement

	Included	Excluded
Population	Male breast cancer patients in the Arab region	Female patients, other populations
Interventions	None	None
Comparators	None	None
Study design	Prospective, retrospective cohort, case reports, and research articles in any language	Reviews, books, protocols, guidelines, and animal studies
Primary outcome	Subtypes in MBC	Irrelevant
Secondary outcomes	Genetic variation, clinicopathological data	Irrelevant

*Abbreviations*: *PICOS* Population, Intervention, Comparator, Outcomes, and Study design, *MBC* Male breast cancer

## Results

### Search outcome

The database search identified a total of 9,337 records, including 3,970 from PubMed, 2,781 from Embase, and 2,586 from Scopus. After the removal of 3,833 duplicates, 5,504 records were screened based on titles and abstracts. During this stage, 5,048 records were excluded because they were reviews, books, guidelines, trial registry records, preclinical studies, or conducted outside Arab countries (Fig. 1).

A total of 456 full-text articles, all of which focused on Arab populations, were assessed for eligibility after the initial title and abstract screening. Of these, 402 studies were excluded due to inclusion of female patients only, conference abstracts without full manuscripts, lack of access to full text, or failure to address genetic characteristics or molecular subtypes. Ultimately, 54 studies met the inclusion criteria and were included in the qualitative synthesis. Across these studies, a total of 1,534 MBC patients were reported and included in the final analysis. A summary of the included studies is presented in Table 2.

**Table 2:** Description of the included articles on MBC subtypes in the Arab population

Reference	Population	Study type	Sample size	Reported subtypes
Alsayed, 2019 [16]	Bahrain, Saudi Arabia	Case report	1	ER, PR, HER2 status reported, Triple-negative
Kridis, 2023 [17]	Tunisia	Cohort study	32	ER, PR, HER2 status reported
El Fouhi, 2020 [18]	Morocco	Cohort study	25	ER, PR, HER2 status reported
Al-Naqqash, 2025 [19]	Iraq	Cohort study	23	ER, PR, HER2 status reported
Manai, 2019 [20]	Tunisia	Cohort study	96	ER, PR, HER2 status reported Luminal A, luminal B, HER2, triple-negative
Abdeljaoued, 2017 [21]	Tunisia	Cross-sectional	130	ER, PR, HER2 status reported, Luminal A, luminal B, HER2, triple-negative
Al Awayshih, 2019 [22]	Jordan	Case report	1	ER, PR, HER2 status reported. Triple-negative
Merad, 2024 [23]	Algeria	Cross-sectional	25	ER, PR, HER2 status reported, Luminal A, luminal B, HER2, triple-negative
Slimani, 2016 [24]	Morocco	Case series	140	ER, PR, HER2 status reported
El Fouhi, 2022 [25]	Morocco	Cross-sectional	21	ER, PR, HER2 status reported, Luminal A, luminal B, HER2, triple-negative
Alouani, 2020 [26]	Morocco	Case report	1	ER, PR status reported
Laabadi, 2013 [27]	Morocco	Case series	6	ER, PR status reported
Elshafiey, 2011 [28]	Egypt	Cohort study	32	ER, PR status reported
Gilbert, 2011 [29]	Egypt & Morocco	Cohort study; multicenter	343; E (211), M (132)	ER, PR, HER2 status reported, GV
Hali, 2011 [30]	Morocco	Case series	20	ER, PR status reported
El-Habbash, 2009 [31]	Libya	Cohort study	22	ER, PR status reported
Soliman, 2014 [32]	Egypt	Cohort study	69	ER, PR status reported
Tawil, 2012 [33]	Lebanon	Case series	47	ER, PR, HER2 status reported
Guaoua, 2014 [34]	Morocco	Case report	1	ER, PR, HER2 status reported, GV
Omari-Alaoui, 2002 [35]	Morocco	Case series	71	ER, PR status reported
Al-Saleh, 2012 [36]	Kuwait	Case report	1	ER, PR status reported
Al-Saleh, 2011 [37]	Kuwait	Case report	1	None
Darouassi, 2010 [38]	Morocco	Case report	1	ER, PR, HER2 status reported
El Harroudi, 2010 [39]	Morocco	Case report	1	None
Troudi, 2007 [40]^b^	Tunisia	Cohort Study	1	ER, PR status reported
Safini, 2016 [41]	Morocco	Case report	1	ER, PR, HER2 status reported
Eshaqi, 2024 [42]	Sultanate of Oman	Case series	2	ER, PR, HER2 status reported
Laham, 2022 [43]	Syria	Case report	1	ER, PR, HER2 status reported
Sakhri, 2024 [44]	Tunisia	Case report	1	ER, PR, HER2 status reported, triple-negative
Sekal, 2014 [45]	Morocco	Case report	1	ER, PR, HER2 status reported, triple-negative
Marafi, 2020 [46]	Kuwait	Case report	1	None
Marzogi, 2025 [47]	Saudi Arabia	Case report	1	ER, PR, HER2 status reported, GV
Sellal, 2011 [48]	Morocco	Case series	21	ER, PR, HER2 status reported
El-Baradie, 2012 [49]	Egypt	Cohort Study	123	ER, PR, HER2 status reported, Luminal A, luminal B, HER2, triple-negative
Al Salloom, 2015 [50]	Saudi Arabia	Case report	1	ER, PR, HER2 status reported
Aldossary, 2019 [51]	Saudi Arabia	Case report	1	ER, PR, HER2 status reported, Triple-positive
Alharbi, 2022 [52]	Saudi Arabia	Case report	1	None
Hammedi, 2010 [53]	Tunisia	Case report	1	ER, PR, HER2 status reported
Bourhafour, 2011 [54]	Morocco	Cohort Study	127	ER, PR status reported
Ekram, 2023 [55]	Saudi Arabia	Case report	1	GV
Jaheddine, 2024 [56]	Morocco	Case report	1	ER, PR, HER2 status reported
Temmim, 2001 [57]	Kuwait	Cohort study	41	ER, PR status reported
El-Beshbeshi, 2012 [58]	Egypt	Case series	37	ER, PR status reported
Tahrir, 2022 [59]	Morocco	Case report	1	ER, PR, HER2 status reported, Triple negative
Zebbakh, 2024 [60]	Morocco	Case report	1	ER, PR, HER2 status reported, GV
Soliman, 2016 [61]	Egypt	Cohort study	39	ER, PR status reported
Tarrab, 2021 [62]	Syria	Case report	1	ER, PR, HER2 status reported
Al Zouman, 2010 [63]	Saudi Arabia	Case report	1	ER, PR status reported
Kridis-Rejeb, 2020 [64]	Tunisia	Case series	6	ER, PR, HER2 status reported, GV
Awadelkarim, 2007 [65]^b^	Italy^a^	Cross-sectional	1	GV
Hamdi, 2021 [66]^b^	Tunisia	Cross-sectional	5	ER, PR, HER2 status reported, GV
Sakhri, 2019 [67]	Tunisia	Case series	3	ER, PR status reported
Taha, 2013 [68]	Morocco	Case report	1	None
Kallel, 2014 [69]	Tunisia	Case report	1	ER, PR, HER2 status reported
Total (*n*)			1,534	

*Abbreviations*: *ER* Estrogen receptor, *PR* Progesterone receptor, *HER2* Human epidermal growth factor receptor 2, *GV* Genetic variation

^a^Study conducted in Italy; included patients were of Sudanese origin

^b^Cross-sectional studies including both male and female patients; only male breast cancer cases were extracted and reported in this review

### Quality of eligible articles

**Fig. 2:**
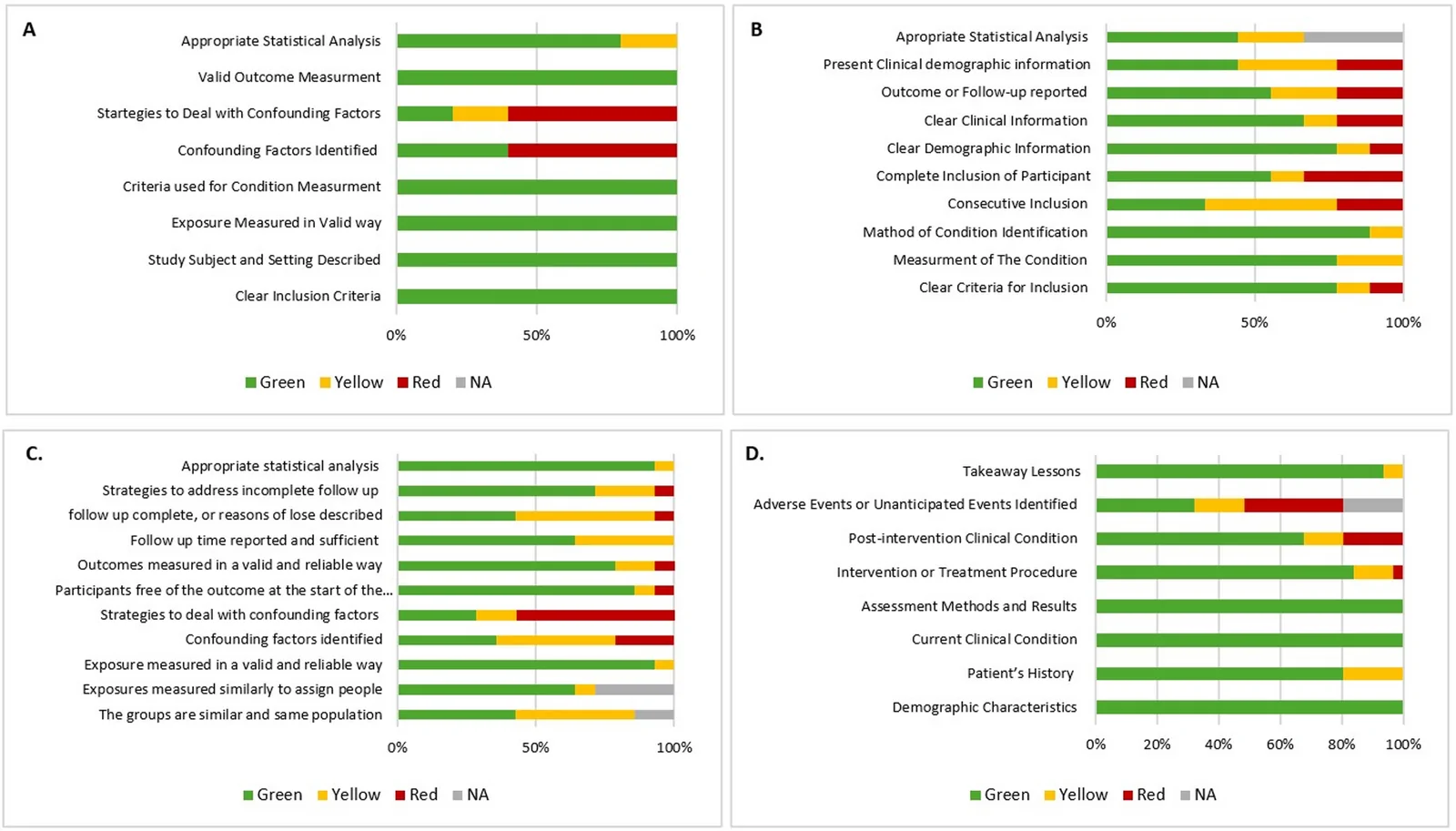
Distribution of risk of bias judgments for included studies assessed using the Joanna Briggs Institute (JBI) critical appraisal checklists according to study design. Panels show: **A** cross-sectional studies (*n* = 5), **B** case series (*n* = 9), **C** cohort studies (*n* = 14), and (**D**) case reports (*n* = 26). Bars represent the proportion of studies rated as low risk of bias (green), unclear risk (yellow), high risk (red), or not applicable (NA) (grey) for each appraisal item

Risk of bias was assessed in the 54 included studies using design-specific JBI critical appraisal checklists, including 26 case reports (48.1%), 14 cohort studies (25.9%), 9 case series (16.7%), and 5 cross-sectional studies (9.3%). Based on the JBI critical appraisal checklists for each study design, 26 studies (48.1%) were considered to have low risk of bias, 28 studies (51.9%) moderate risk of bias, and none were judged to have high risk of bias, indicating that the overall methodological quality of the included studies was moderate (Fig. 2).

Across all study designs, most domains were rated as low risk of bias, particularly those related to clear inclusion criteria, valid measurement of exposure and outcomes, and appropriate statistical analysis, with 70–90% of studies receiving low-risk ratings for these items. For cross-sectional studies, 100% demonstrated low risk for inclusion criteria, exposure measurement, and condition measurement, whereas 60–80% showed high or unclear risk for identification and management of confounding factors.

In case series, low risk of bias was observed in 75–90% of studies for case definition, condition measurement, and method of case identification; however, around 40–55% were rated as high or unclear risk for consecutive inclusion, complete inclusion of participants, and outcome follow-up reporting. Cohort studies demonstrated low risk of bias in 70–85% of studies for exposure and outcome measurement and statistical analysis, but 50–65% showed high or unclear risk regarding identification and control of confounding and 30–40% for completeness of follow-up and baseline group similarity.

Case reports exhibited the most favorable risk-of-bias profile, with approximately 80–95% of studies rated as low risk for reporting of demographics, patient history, clinical condition, diagnostic assessment, and intervention details, although adverse events and post-intervention outcomes were incompletely reported in approximately 30–40% of reports. The distribution of study quality is shown in Fig. 2. Detailed item-level risk-of-bias assessments for each study and study design are provided in Supplementary Table S3.

### Participant characteristics

Among the 1,534 MBC patients included in this systematic review, histopathological subtype was reported for 1,450 patients. Among these, invasive ductal carcinoma (IDC) was the predominant histopathological subtype, accounting for 1,342 cases (92.6%), while other subtypes such as lobular (20; 1.4%), papillary (13; 0.9%), mucinous (4; 0.3%), and ductal carcinoma in situ (DCIS) (9; 0.6%) were comparatively rare (Table 3). Tumor size, pathological stage, tumor grade, nodal status, and metastatic status were reported for the overall cohort of 1,534 patients. Tumor size was most frequently reported as T2 (297 patients; 19.3%) and T4 (284 patients; 18.5%), followed by T3 **(**144; 9.4%) and T1 (84; 5.5%); however, tumor size was either unknown or not reported in 356 patients (23.2%) (Table 4). Stage III disease was the most common (303 patients; 19.8%), followed by Stage IV **(**212; 13.8%) and Stage II (188; 12.3%), while early-stage disease (Stage I) accounted for 7.6% (117) of cases.

**Table 3:** Histopathological subtypes of the 1,450 male breast cancer patients

Variable	No.	%
Histological subtype		
Ductal	1,342	92.57
Lobular	20	1.40
Mixed (ductal and lobular)	5	0.35
Papillary	13	0.90
Micropapillary	2	0.13
DCIS	9	0.60
Mucinous	4	0.30
Adenocarcinoma	7	0.50
Neuroendocrine carcinoma	1	0.06
Sarcoma	2	0.13
Benign	1	0.06
Others carcinoma	41	2.80
Not reported	3	0.20
Total	1,450	100

*DCIS* Ductal Carcinoma in Situ

**Table 4:** Tumor characteristics and pathological staging among 1,534 male breast cancer patients

Variable	No.	%	Variable	No.	%
Tumor size			**Tumor grade**		
T0	3	0.2	G1	83	5.4
T1	84	5.5	G2	724	47.2
T2	297	19.3	G3	187	12.1
T3	144	9.4	Unknown	67	4.4
T4	284	18.5	Not reported	194	12.6
Unknown	29	1.9	**Nodal status**		
Not reported	327	21.3	N+	624	40.7
Tumor stage			N-	518	33.8
0	1	0.1	N1	233	15.2
I	117	7.6	N2	153	10.0
II	188	12.3	N3	84	5.5
III	303	19.8	Unknown	85	5.5
IV	212	13.8	Not reported	61	4.0
Unknown	50	3.3			
Not reported	382	24.9			
Metastasis					
M0	667	43.5			
M1	229	14.9			
Unknown	29	1.9			
Not reported	258	16.8			

Tumor grades from different classification systems (including Scarff–Bloom–Richardson and Van Nuys) were standardized into three unified categories (G1–G3) to ensure consistency across studies. Histological stage was similarly harmonized across staging systems (including AJCC and UICC classifications). When data were originally reported as percentages, absolute numbers were recalculated based on the total study population. Percentages shown in this table were calculated using the total number of patients (*n* = 1,534). Reporting completeness varied across studies. The combined proportion of unknown and not reported data was 23.2% for tumor size (356/1,534), 28.2% for tumor stage (432/1,534), 17.0% for tumor grade (261/1,534), 18.7% for metastatic status (287/1,534), and 9.5% for nodal state (146/1,534)

Regarding tumor grade, grade 2 (G2) tumors were most prevalent (724 patients; 47.2%), followed by G3 (187; 12.1%**)** and G1 (83; 5.4%). Nodal involvement was reported as positive (N+) in 624 patients (40.7%) and negative in (518; 33.8%). Distant metastases were documented in 229 patients (14.9%), while 667 (43.5%) were reported as non-metastatic at diagnosis; metastatic status was unknown or not reported in 287 cases (18.7%). Notably, there was substantial heterogeneity and incomplete reporting across studies, particularly for tumor size, pathological stage, nodal status, and metastatic status.

### Intrinsic subtypes

**Fig. 3:**
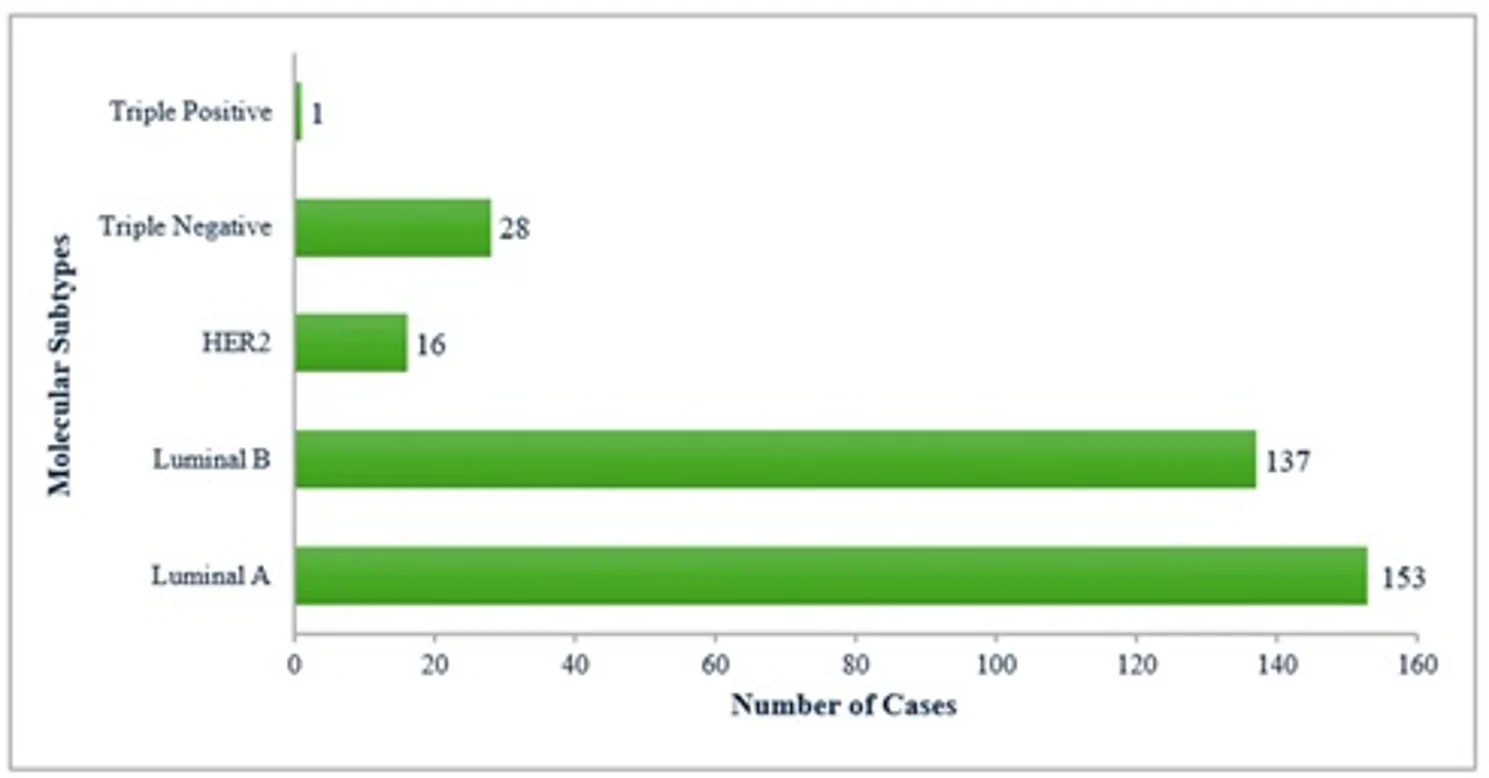
Distribution of intrinsic molecular subtypes among male breast cancer patients. Bars represent the number of patients classified into each intrinsic molecular subtype (luminal A, luminal B, HER2-positive. Triple-negative, and triple-positive) among studies that reported molecular subtype data (*n* = 335)

A total of 335 patients were reported in articles that provided molecular subtype classification. Overall, luminal subtypes predominated (Table 5 and Fig. 3). Luminal A was the most frequently reported subtype, comprising 153 cases (45.7%), followed by luminal B, accounting for 137 cases (40.9%). Together, luminal tumors represented approximately 86% of all cases with available subtype data. The predominance of luminal disease was consistent across larger cohorts, including studies by Manai et al. [20] and Abdeljaoued et al. [21], in which luminal A and luminal B subtypes collectively constituted the vast majority of cases.

**Table 5:** Distribution of intrinsic subtypes of MBC across articles categorized by subtype (luminal A, luminal B, HER2+, triple-negative, triple-positive) and number of participants, expressed as counts and percentages (%)

Ref.	No. of participants	Luminal A *n* (%)	Luminal B *n* (%)	HER2+ *n* (%)	Triple-negative *n* (%)	Triple-Positive *n* (%)
[16]	1				1 (100)	
[20]	96	51 (53.1)	40 (41.7)	2 (2.1)	3 (3.1)	
[21]	130	59 (45.4)	58 (44.6)	6 (4.6)	7 (5.4)	
[22]	1				1 (100)	
[23]	25	3 (12.0)	20 (80.0)	1 (4.0)	1 (4.0)	
[25]	21	2 (9.5)	16 (76.2)	1 (4.8)	2 (9.5)	
[44]	1				1 (100)	
[45]	1				1 (100)	
[49]	57	38 (66.7)	3 (5.3)	6 (10.5)	10 (17.5)	
[51]	1					1 (100)
[59]	1				1 (100.0)	
Total (%)	335	153 (45.7)	137 (40.9)	16 (4.8)	28 (8.3)	1 (0.3)

HER2-positive tumors were relatively uncommon, with 16 cases (4.8%) reported overall. Similarly, the triple-negative subtype accounted for 28 cases (8.4%), remaining a minority across studies but showing variability between cohorts. In contrast, the triple-positive subtype was rare, with only one case (0.3%) reported across all included articles. Overall, substantial variability was observed in subtype distribution across studies, particularly in smaller cohorts; however, the consistent predominance of luminal subtypes across larger series highlights the hormone receptor–driven biology of MBC.

### Reported receptor status

Hormone receptor status was reported for a subset of the included studies (*n* = 1,031 MBC patients; Table 6). ER positivity (ER⁺) was observed in 737/1,031 patients (71.5%), with reported frequencies ranging from 33.3% to 100% across studies. Large retrospective cohorts such as Manai et al. (*n* = 96) [20], Abdeljaoued et al. (*n* = 130) [21], and Silmani et al. (*n* = 140) [24] consistently demonstrated ER⁺ rates exceeding 85%, whereas smaller single-center studies and case reports showed greater variability in receptor expression.

**Table 6:** Distribution of receptor status of MBC across articles categorized by receptors (ER, PR, and HER2) and number of participants, expressed as counts and percentages (%)

Receptor status									
Ref.	Tested (*n*)^*^	HER2 Tested (*n*)^†^	ER+ *n* (%)	ER- *n* (%)	PR+ *n* (%)	PR- *n* (%)	HER2+ *n* (%)	HER2- *n* (%)	HER2 equivocal *n* (%)
[16]	1	1	–	1 (100)	–	1(100)	–	1(100)	–
[17]	32	32	32 (100)	–	32 (100)	–	30 (93.8)	2 (6.3)	–
[18]	18	NR	15 (83.3)	3 (16.7)	15 (83.3)	3 (16.7)	NR	NR	–
[19]	18	17	16 (88.9)	2 (11.1)	16 (88.9)	2 (11.1)	4 (23.5)	11 (64.7)	2 (11.8)
[20]	96	96	91 (94.8)	5 (5.2)	81 (84.4)	15 (15.6)	6 (6.2)	90 (93.8)	–
[21]	130	130	118 (90.8)	12 (9.2)	108 (83.1)	22 (16.9)	10 (7.7)	120 (92.3)	–
[22]	1	1	–	1 (100)	–	1 (100)	–	1 (100)	–
[23]	25	25	23 (92.0)	2 (8.0)	23 (92.0)	2 (8.0)	1 (4.0)	24 (96.0)	
[24]	140	140	125 (89.3)	15 (10.7)	80 (57.1)	60 (42.9)	–	140 (100)	–
[25]	21	NR	19 (90.5)	2 (9.5)	19 (90.5)	2 (9.5)	NR	NR	–
[26]	1	NR	1 (100)	–	1 (100)	–	NR	NR	–
[27]	6	3	6 (100)	–	5 (83.3)	1 (16.7)	–	3 (100)	–
[28]	18	NR	15 (83.3)	3 (16.7)	14 (77.8)	4 (22.2)	NR	NR	
[29]	124	NR	103 (83.1)	21 (16.9)	92 (76.0)	29 (24.0)	NR	NR	–
[30]	1	NR	1 (100)	–	1 (100)	–	NR	NR	–
[31]	11	NR	8 (72.7)	3 (27.3)	8 (72.7)	3 (27.3)	NR	NR	
[32]	69	NR	29 (42.0)	40 (58.0)	29 (42.0)	40 (58.0)	NR	NR	–
[33]	34	32	32 (94.1)	2 (5.9)	22 (64.7)	12 (35.3)	3 (9.4)	19 (59.4)	10 (31.2)
[34]	1	1	1 (100)	–	1 (100)	–	–	1 (100)	–
[35]	5	NR	4 (80.0)	1 (20.0)	4 (80.0)	1 (20.0)	NR	NR	–
[36]	1	NR	–	1 (100)	–	1 (100)	NR	NR	–
[38]	1	1	–	1 (100)	1 (100)	–	–	1 (100)	–
[40]	1	NR	1 (100)	–	1 (100)	–	NR	NR	–
[41]	1	1	1 (100)	–	1 (100)	–	–	1 (100)	–
[42]	2	1	2 (100)	–	1 (50.0)	1 (50.0)	1 (100)	–	–
[43]	1	1	1 (100)	–	1 (100)	–	–	1 (100)	–
[44]	1	1	–	1 (100)	–	1 (100)	–	1 (100)	–
[45]	1	1	–	1 (100)	–	1 (100)	–	1 (100)	–
[47]	1	1	1 (100)	–	–	1 (100)	1 (100)	–	–
[48]	20	3	17 (85.0)	3 (15.0)	17 (85.0)	3 (15.0)	–	3 (100)	–
[49]	91	57	65 (71.4)	26 (28.6)	63 (69.2)	28 (30.8)	6 (10.5)	48 (84.2)	3 (5.3)
[50]	1	1	1 (100)	–	1 (100)	–	–	1 (100)	–
[51]	1	1	1 (100)	–	1 (100)	–	1 (100)	–	–
[53]	1	1	–	1 (100)	1 (100)	–	–	1 (100)	–
[54]	61	NR	39 (63.9)	22 (36.1)	39 (63.9)	22 (36.1)	NR	NR	–
[56]	1	1	–	1 (100)	–	1 (100)	1 (100)	–	–
[57]	18	18	16 (88.9)	2 (11.1)	11 (61.1)	7 (38.9)	9 (50.0)	9 (50.0)	–
[58]	21	NR	18 (85.7)	3 (14.3)	18 (85.7)	3 (14.3)	NR	NR	–
[59]	1	1	–	1 (100)	–	1 (100)	–	1 (100)	–
[60]	1	1	1 (100)	–	1 (100)	–	–	1 (100)	–
[61]	38	NR	31 (81.6)	7 (18.4)	31 (81.6)	7 (18.4)	NR	NR	–
[62]	1	1	1 (100)	–	–	1 (100)	–	1 (100)	–
[63]	1	NR	1 (100)	–	1 (100)	–	NR	NR	–
[64]	6	6	4 (66.7)	2 (33.3)	6 (100)	–	–	6 (100)	–
[66]	2	2	2 (100)	–	1 (50.0)	1 (50.0)	–	2 (100)	–
[67]	3	NR	1 (33.3)	2 (66.7)	1 (33.3)	2 (66.7)	NR	NR	–
[69]	1	1	1 (100)	–	1 (100)	–	–	1 (100)	–
Total (%)	1,038	579	737 (71.5)	187 (18.1)	738 (71.6)	279 (27.1)	73 (12.6)	491 (84.8)	15 (2.59)

*Abbreviations*: *ER* Estrogen receptor, *PR* Progesterone receptor, *HER2* Human epidermal growth factor receptor 2, *UND* Undetermined, *NR* Not reported

^*^Tested (*n*) refers to the number of patients with available ER and/or PR results. Percentages for ER-positive, ER-negative, PR-positive, and PR-negative cases were calculated using this denominator

^†^HER2 tested (*n*) refers to the number of patients evaluated for HER2. Percentages for HER-positive, HER2-negative, and HER2-equivocal cases were calculated using the number of HER2-tested patients as the denominator

PR positivity (PR⁺) was reported in 738/1,031 patients (71.6%), with study-specific frequencies ranging from 33.3% to 100%. Concurrent ER⁺/PR⁺ expression predominated across most cohorts, while lower PR⁺ rates were more commonly observed in smaller samples and older studies with limited receptor testing.

HER2 status was less consistently reported and demonstrated greater heterogeneity. HER2 positivity was documented in 73 patients (12.6%), with reported proportions ranging from 4.0% to 100% in studies that assessed HER2 expression. Several studies evaluated HER2 status in only a subset of patients or did not report HER2 testing at all, reflecting variability in testing availability and reporting practices over time. Overall, larger cohort studies exhibited more stable hormone receptor expression patterns, whereas smaller series and case reports showed wider variability (Table 6).

### Genetic characteristics of MBC

Genetic alterations were reported in a limited number of included studies and were investigated using a range of methodologies, including immunohistochemistry, Sanger sequencing, targeted next-generation sequencing, multigene panel sequencing, and whole-exome sequencing.

Most pathogenic variants were most frequently identified in *BRCA2*, supporting its prominent role in hereditary MBC. Recurrent pathogenic *BRCA2* variants, including c.17–20delAAGA (p. Lys6Xfs), c.6406_6407delTT, c.6428 C > A (p. Ser2143Ter), c.1310_1313delAAGA (p. Lys437fs*22), and c.1389_1390delAG (p. Val464fs*3), were reported across independent cohorts [34, 64–66].

Two studies reported no pathogenic *BRCA* or *BRCA2/CDH1* variants following genetic testing [47, 60], while one study assessed *BRCA2* protein expression by immunohistochemistry rather than DNA sequencing, reporting wildtype protein expression in 71.1% of cases and non-wild type expression in 28.9% [29]. These findings highlight the heterogeneity of genetic susceptibility in MBC and suggest that not all cases are attributable to known high-penetrance mutations.

Beyond *BRCA2*, a pathogenic *CHEK2* stop-gain variant (c.636T > G (p.Tyr212Ter) was identified in a single patient, supporting the involvement of non-*BRCA* hereditary cancer genes in a subset of cases [55]. Additionally, whole-exome sequencing performed in one early-onset *BRCA*-negative patient identified candidate variants in *MSH5*,* DCC*,* ERBB3*,* NOTCH3*,* DIAPH1*, and *DNAH11*; however, these variants were considered exploratory, and their pathogenic significance has not been established [64] (Table 7).

**Table 7:** Overview of genetic mutations reported in 59 MBC patients

Ref.	No. tested	Genes	Genetic testing methodology	Alteration Status	HGVS Variant Annotation	Exon
[29]	45	*BRCA2*	Immunohistochemistry (IHC)	Wild type	NR	NR
[34]	1	*BRCA2*	PCR amplification and bidirectional Sanger sequencing	Pathogenic germline variant detected	*BRCA2* c.6428 C > A (p. Ser2143Ter)	11
[47]	1	*BRCA*	NR	Negative	NR	NR
[55]	1	*CHEK2*	Multigene panel testing using next-generation sequencing (NGS)	Pathogenic germline variant detected	*CHEK2* c.636T > G(p. Tyr212Ter)	NR
[60]	1	*BRCA2*,* CDH1*	NR	No pathogenic *BRCA2/CDH1* variant detected	NR	NR
[64]	6	*BRCA1*,* BRCA2*	Targeted *BRCA1/2* sequencing + Sanger confirmation	Pathogenic germline variant detected	*BRCA2* c.17_20delAAGA (p. Lys6Xfs)	2
[64]^a^	1	*MSH5*,* DCC*,* ERBB3*,* NOTCH3*,* DIAPH1*,* DNAH11*	Whole-exome sequencing	Candidate variants detected	NR	NR
[65]	1	*BRCA2*	Protein truncation test (PTT), denaturing high-performance liquid chromatography (DHPLC), and direct sequencing	Pathogenic germline variant detected	*BRCA2* c.6406_6407delTT	NR
[66]	2	*BRCA2*	*BRCA* testing using Sanger sequencing and/or NGS	Pathogenic germline variants detected	*BRCA2* c.1310_1313delAAGA (p. Lys437fs^*^22); *BRCA2* c.1389_1390delAG (p. Val464fs^*^3)	10
Total (*n*)	59					

*Abbreviations*: *BRCA2* Breast cancer susceptibility gene 2. *CDH1* Cadherin (1) *CHEK2* Checkpoint kinase (2) *MSH5* MutS homolog 5, *DCC* Deleted in colorectal carcinoma, *ERBB3* Erb-B2 receptor tyrosine kinase 3, *NOTCH3* Notch receptor 3, *DIAPH1* Diaphanous-related formin 1, *DNAH11* Dynein axonemal heavy chain 11, *DHPLC* Denaturing high-performance liquid chromatography, *IHC* immunohistochemistry, *NGS* Next-generation sequencing, *NR* Not reported

^a^Candidate variants identified using exome/panel sequencing; pathogenicity was not established

### Treatment patterns

**Fig. 4:**
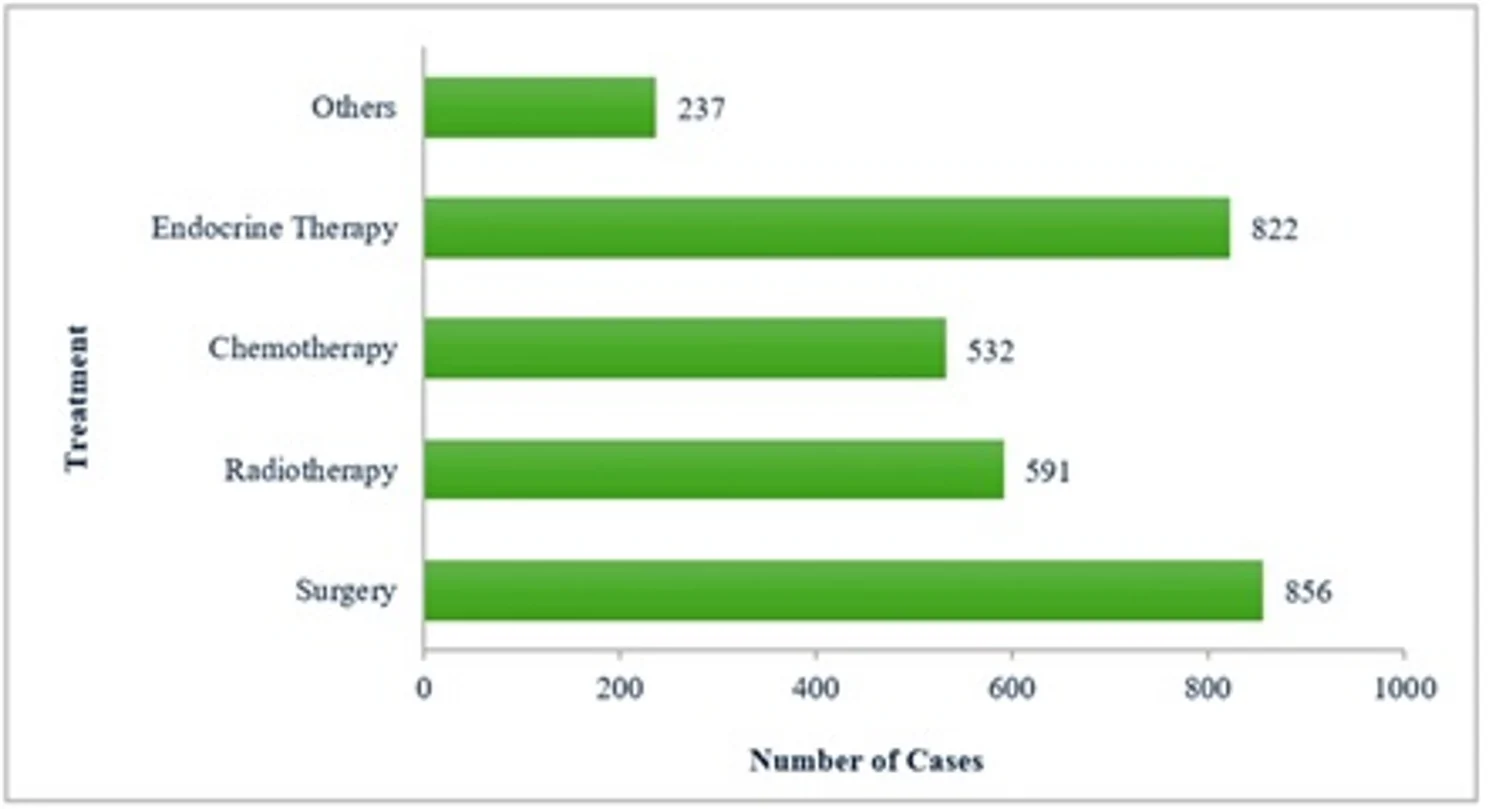
Distribution of treatment modalities among male breast cancer patients. Bars represent the number of patients reported to have received surgery, radiotherapy, chemotherapy, endocrine therapy, or other treatments. Treatment categories are not mutually exclusive because individual patients may have received more than one treatment modality. “Other treatments” include therapies that were infrequently reported or did not fall within the major treatment categories

As summarized in Fig. 4 and Table 8, reported treatment strategies for MBC included surgery, radiotherapy, chemotherapy, endocrine therapy, as well as combined and supportive treatment strategies. Surgical management was the most frequently reported modality, administered to 856 patients. Mastectomy-based procedures were predominant, especially radical mastectomy (227; 26.5%), mastectomy with axillary dissection (178; 20.8%), and modified radical mastectomy (177; 20.7%). Procedures combining modified radical mastectomy with axillary dissection accounted for 10.8% (92) of cases. Breast-conserving surgery, including lumpectomy and quadrantectomy, was infrequently reported (20; 2.3%), while 7.7% (66) of patients did not undergo surgical intervention.

**Table 8:** Overview of treatments used in MBC patients

Treatment	Number of patients (%)	References
Surgery (*n* = 856)		
Simple mastectomy	16 (1.9)	[27, 28, 35–38, 42, 49, 58, 69]
Mastectomy with axillary dissection (SLNB or ALND)	178 (20.8)	[18, 24, 26, 30, 31, 39, 59, 62, 67, 68]
Modified radical mastectomy (MRM)	177 (20.7)	[16, 25, 27, 28, 35, 49, 54, 58, 64]
MRM + axillary dissection	92 (10.7)	[19, 22, 32, 50, 51, 55]
Radical mastectomy	227 (26.5)	[17, 28, 35, 48, 49, 52, 54, 58]
Radical mastectomy with axillary dissection (SLNB or ALND)	22 (2.6)	[23, 43, 53]
Total mastectomy (no axillary dissection)	11(1.3)	[54]
Conservative surgery (lumpectomy/quadrantectomy)	20 (2.3)	[19, 24, 31, 34, 35, 48, 49, 54, 58, 61]
Debulking surgery	1 (0.1)	[63]
Biopsy only	40 (4.7)	[23, 28, 31, 49]
No surgery	66 (7.7)	[16, 24, 27, 28, 41, 44, 48, 58]
Surgery not reported	3 (0.35)	[45–47]
Other	3 (0.35)	[19]
Radiotherapy (*n* = 591)		
Radiotherapy	345 (58.4)	[18, 19, 24–26, 31, 32, 34, 35, 38, 44, 48, 53, 54, 61–64, 67]
Adjuvant	143 (24.2)	[17, 28, 39, 49, 52, 54, 55, 58, 59]
Palliative	28 (4.7)	[28, 49]
No radiotherapy	38 (6.4)	[28, 49, 58]
Radiotherapy not specified	37 (6.3)	[49]
Chemotherapy (*n* = 532)		
Chemotherapy	189 (35.5)	[18, 19, 25, 27, 34, 44, 53, 54, 61–63]
Adjuvant	232 (43.6)	[17, 24, 27, 28, 32, 38, 39, 49, 52, 54, 55, 58, 59, 64, 69]
Neoadjuvant	18 (3.4)	[24, 27, 54]
Palliative	12 (2.3)	[28, 47, 49]
No chemotherapy	40 (7.5)	[28, 49, 58]
Chemotherapy not specified	41 (7.7)	[49]
Endocrine therapy (*n* = 810)		
Endocrine therapy (General)	257 (31.8)	[18, 19, 24–26, 48, 49, 54, 56]
Tamoxifen	69 (8.5)	[17, 41, 53, 55, 61, 64, 67]
Adjuvant Endocrine therapy	147 (18.15)	[28, 35, 54, 58]
Palliative Endocrine therapy	15 (1.85)	[28, 35]
No Endocrine therapy	26 (3.2)	[49, 58]
Endocrine therapy not specified	65 (8)	[49]
Endocrine therapy not reported	231 (28.5)	—
Targeted therapy (*n* = 12)		
Trastuzumab / Herceptin	12 (100)	[17, 25]
Others (*n* = 248)		
Chemotherapy + radiotherapy	45 (18.1)	[29, 30, 48]
Surgery + Hormone therapy	115 (46.5)	[29]
Best supportive care	11 (4.4)	[54]
Studies not reporting treatment	77 (31)	[20, 22, 23, 33, 36, 40, 42, 43, 57, 60, 65, 66, 68]

Treatment categories are presented as reported in the original studies. “Endocrine therapy (general)” indicates that a study mentioned endocrine therapy without further detail, whereas “Endocrine therapy not specified” refers to studies that explicitly stated that the type was not specified

*Abbreviations*: *MRM* Modified radical mastectomy, *ALND* Axillary lymph node dissection, *SLNB* Sentinel lymph node biopsy, *RT* Radiotherapy, *CT* Chemotherapy, *HT* Hormonal therapy

Radiotherapy was reported in 591 patients, most delivered as definitive radiotherapy (345; 58.4%) or in the adjuvant setting (143; 24.2%). Palliative radiotherapy was reported in 4.7% (28) of cases, whereas 6.4% (38) of patients did not receive radiotherapy and 6.3% (37) had unspecified radiotherapy status. Chemotherapy was administered to 532 patients, predominantly as adjuvant treatment (232; 43.6%), followed by definitive chemotherapy (189; 35.5%). Neoadjuvant and palliative chemotherapy were reported in 3.4% (18) and 2.3% (12) of patients, respectively, while 7.5% (40) did not receive chemotherapy and 7.7% (41) had unspecified chemotherapy status.

Endocrine therapy was reported in 810 patients, with endocrine treatment of any type administered in 31.7% (257) of cases. Adjuvant endocrine therapy was the most frequently reported (147; 18.1%), with tamoxifen being the predominant agent (69; 8.5%). A proportion of patients did not receive endocrine therapy (26; 3.2%), and endocrine treatment status was either not reported (231; 28.5%) or unspecified (65; 8.0%) in several studies. HER2-targeted therapy with trastuzumab was infrequently reported in only 12 patients. Combined treatment approaches were also documented, including surgery with endocrine therapy (115; 46.4%) and combined chemotherapy and radiotherapy (45; 18.1%). Best supportive care alone was reported in 4.4% (11) of patients.

## Discussion

This systematic review updates the evidence on molecular subtypes, receptor status, genetic alterations, and treatment patterns of MBC in Arab populations. Using a comprehensive multi-database search through 28 of December 2025, 54 studies comprising 1,534 patients were identified, reflecting both increased research output and continued reliance on small, heterogeneous cohorts. Although this represents broader coverage than earlier regional reviews, molecular subtyping and genetic testing were reported in only a subset of studies. Unlike the previous Arab-world systematic review published in February 2022 [11], which focused on epidemiologic and clinicopathologic features, the present review specifically addresses molecular subtype distribution and genetic alterations and incorporates more recent literature.

In this review, intrinsic subtype distribution in Arab MBC was strongly skewed toward luminal disease, reinforcing the concept that MBC in this region is predominantly hormone receptor driven. The clear predominance of luminal A and luminal B subtypes across studies, particularly in larger cohorts such as those by Manai et al. and Abdeljaoued et al. [20, 21], suggests a relatively conserved biological profile characterized by endocrine responsiveness. In contrast, HER2-positive and triple-negative tumors were consistently infrequent, while triple-positive disease was exceptionally rare, indicating that biologically aggressive, receptor-poor phenotypes represent a minority of MBC cases in this population.

This subtype distribution mirrors patterns reported in FBC from the region and beyond, where luminal A and B predominate, including Egyptian cohorts [70], and in global data identifying luminal A as the most common subtype overall [71]. These findings suggest shared hormone-driven biology across sexes, although relative proportions may vary by region and diagnostic practices. Clinically, the dominance of luminal subtypes highlights the central role of endocrine therapy in MBC, while the rarity of HER2-positive and triple-negative disease suggest that only a small proportion of patients are likely to benefit from HER2-targeted therapies or emerging immunotherapeutic approaches. These findings are also consistent with large international MBC cohorts, which similarly report a predominance of hormone receptor-positive luminal tumors, suggesting that the hormone-dependent biology of MBC is broadly similar across different populations despite geographic variation [72].

Consistent with the predominance of luminal disease, hormone receptor expression was highly prevalent across the included Arab studies. Although receptor expression patterns were generally similar in the larger cohorts, greater variability was observed among smaller studies, likely reflecting differences in testing practices, assay availability, and reporting standards rather than true biological difference. HER2-positive disease remained uncommon in our analysis, consistent with previous reports [73]. These findings emphasize the importance of standardized and comprehensive assessment of ER, PR, HER2 and Ki-67 to enable accurate molecular subtype classification, facilitate meaningful comparisons across studies, and support appropriate treatment selection in men with breast cancer.

Studies varied in the level of detail provided, with some reporting only specific intrinsic subtypes, others focusing on receptor status, and a few including both. This variability in data reflects the heterogeneity of practice for MBC in this region and the importance of categorizing cases by subtype to guide personalized treatment strategies and improve patient outcomes. In addition, differences in the availability of immunohistochemistry, HER2 testing, Ki-67 assessment, and genetic sequencing across healthcare systems in the Arab region may have influenced both molecular subtype classification and the detection of genetic alterations. Consequently, some of the observed variation across studies may reflect differences in diagnostic infrastructure and reporting practices rather than true biological differences.

Genetic reporting in Arab-region studies remains limited and heterogeneous. Genetic testing was reported for only 59 of the 1,534 patients included in this review, representing a small proportion of the overall study population. Therefore, the genetic findings should be interpreted with caution. Among the genetically tested patients, pathogenic alteration most commonly involved *BRCA2.* Recurrent *BRCA2* variants, including c.17–20delAAGA (p. Lys6Xfs) and c.6406_6407delTT, were identified across independent studies, indicating that BRCA2 was the most frequently reported susceptibility gene among the limited number of genetically tested MBC cases included in this review [34, 64–66]. Although based on a limited number of genetically tested patients, this finding is consistent with the established role of BRCA2 as the principal hereditary susceptibility gene in MBC and is in accordance with current recommendations advocating germline genetic testing for all men with breast cancer to inform patient management and family counselling [74].

Conversely, several studies reported wild-type or negative *BRCA* findings despite testing, indicating that a proportion of MBC cases are not explained by detectable high-penetrance variants using current approaches [29, 47]. Beyond *BRCA2*, a single pathogenic *CHEK2* stop-gain variant identified by whole-exome sequencing remained unvalidated and of uncertain clinical significance. The diversity of genetic testing methodologies—including immunohistochemistry, Sanger sequencing, targeted next-generation sequencing, and whole-exome sequencing—may have influenced the spectrum of reported variants and limit direct comparison between studies [55, 64].

Treatment selection for MBC is influenced by tumor biology, disease stage, patient age and comorbidities, and therapeutic intent. Here, surgery was the most frequently reported treatment modality, with mastectomy-based procedures predominating. This likely reflects both anatomical considerations in men and the limited availability of MBC-specific evidence, with treatment recommendations continuing to rely largely on evidence extrapolated from FBC. Complete surgical excision remains central to local disease control and is associated with improved survival, particularly when margins are negative [75]. The preference for mastectomy may also be influenced by patient perceptions of surgery as a definitive intervention, especially in settings where access to adjuvant therapies may be variable [76, 77].

Use of radiotherapy and chemotherapy varied considerably across studies, often reflecting differences in disease stage, institutional practice, and reporting completeness. Chemotherapy was primarily administered in the adjuvant setting, while neoadjuvant and palliative use was uncommon, consistent with the predominance of hormone receptor–positive disease observed in this population. Endocrine therapy, most commonly tamoxifen, was reported in a substantial proportion of patients, reinforcing its central role in MBC management given the high rates of ER and PR positivity documented in this review.

Overall, this systematic review advances current understanding of the epidemiologic, molecular, and therapeutic heterogeneity of MBC in Arab populations. By synthesizing data on intrinsic subtypes, receptor status, genetic alterations, and treatment patterns, this study highlights clinically relevant trends and reinforces the importance of routine hormone receptor and *BRCA* testing to support personalized treatment decisions. From a health systems perspective, the findings emphasize the need to strengthen diagnostic infrastructure, standardize molecular characteristics, and expand access to molecular and genetic testing across the region to facilitate equitable implementation of precision oncology.

Nevertheless, our study has limitations. Despite an expanded evidence base compared with earlier regional reviews, the number of eligible studies remains limited, and many relied on small cohorts or case reports. This constrains the generalizability of findings and limits the ability to draw firm conclusions regarding subtype distribution, genetic susceptibility, and treatment outcomes. Furthermore, the available evidence was derived predominantly from studies conducted in Morocco, Tunisia, and Egypt, with several Arab countries remaining underrepresented. Consequently, the findings may not be generalizable to all Arab populations because regional differences in healthcare systems, diagnostic practices, genetic background, and patient characteristics may influence the epidemiological and molecular characteristics of MBC. Selection bias is also possible, as many studies were institution-based and may not reflect the broader male population across Arab countries. In addition, the predominance of retrospective designs limits causal inference and increases vulnerability to confounding.

Substantial heterogeneity in reporting further complicates interpretation. Some studies focused narrowly on specific subtypes, while others lacked complete receptor or genetic data. Among studies reporting genetic findings, testing was performed in only a small subset of patients and employed heterogeneous methodologies, including immunohistochemistry, Sanger sequencing, targeted next-generation sequencing, and whole-exome sequencing. These methodological differences may have influenced variant detection and limit direct comparison of genetic findings across studies. Accordingly, the apparent predominance of BRCA2 should be interpreted cautiously given the limited number of genetically tested patients and the heterogeneity of testing methodologies.

Furthermore, many studies, particularly older publications, did not report Ki-67 expression or apply standardized immunohistochemical surrogate criteria for intrinsic molecular subtype classification. Therefore, intrinsic molecular subtypes were extracted only when explicitly reported, and no attempt was made to reclassify subtypes when the required biomarkers were unavailable. Differences in subtype definitions and reporting practices across studies may therefore have influenced the reported frequencies of intrinsic molecular subtypes, although inclusion of these studies improved the overall completeness of the evidence base.

Although several outcomes were reported as prevalence estimates, a quantitative meta-analysis of proportions was considered but was not performed because the included studies were not sufficiently comparable for meaningful statistical pooling. Variability in study design, patient populations, molecular subtype definitions, completeness of receptor and genetic reporting, and genetic testing methodologies limited the comparability of prevalence estimates and may have resulted in misleading pooled estimates.

Future research should prioritize standardized reporting of molecular subtypes and receptor status, broader implementation of genetic testing, and the development of large, prospective, multicenter studies representative of Arab populations. Standardized application of immunohistochemical surrogate definitions for intrinsic molecular subtypes and harmonized reporting of molecular biomarkers would also improve comparability across future studies. Regional and international collaborations will be essential to improve data quality, reduce disparities in care, and translate emerging molecular insights into evidence-based, region-specific management strategies for MBC.

## Conclusion

This study provides updated insights into the molecular subtypes and genetic characteristics of MBC in Arab populations, a region where evidence remains limited. The findings demonstrate a clear predominance of hormone receptor–positive, luminal subtypes, underscoring the clinical relevance of endocrine-based therapies in this population. Genetic data, although sparse, most frequently identified pathogenic *BRCA2* variants, highlighting the importance of genetic screening in selected MBC patients. Despite addressing an important knowledge gap, the available evidence is derived largely from small and heterogeneous studies, emphasizing the need for larger, well-designed, regionally representative.

## Supplementary Information

Supplementary Material 1.

Supplementary Material 2.

Supplementary Material 3.

## Data Availability

No datasets were generated or analysed during the current study.

## Abbreviations

AJCC: American Joint Committee on Cancer
ALND: Axillary lymph node dissection
BRCA1: Breast cancer susceptibility gene 1
BRCA2: Breast cancer susceptibility gene 2
CDH1: Cadherin 1
CHEK2: Checkpoint kinase 2
CT: Chemotherapy
DCC: Deleted in colorectal carcinoma
DCIS: Ductal carcinoma in situ
DHPLC: DHPLC Denaturing high-performance liquid chromatography
DIAPH1: Diaphanous-related formin 1
DNAH11: Dynein axonemal heavy chain 11
ER: Estrogen receptor
ERBB3: Erb-B2 receptor tyrosine kinase 3
FBC: Female breast cancer
GV: Genetic variation
HER2: Human epidermal growth factor receptor 2
HT: Hormonal therapy
IDC: Invasive ductal carcinoma
IHC: Immunohistochemistry
JBI: Joanna Briggs Institute
MBC: Male breast cancer
MeSH: Medical Subject Headings
MRM: Modified radical mastectomy
MSH5: MutS homolog 5
NGS: Next-generation sequencing
NOTCH3: Notch receptor 3
NR: Not reported
PICOS: Population, Intervention, Comparator, Outcomes, and Study design
PR: Progesterone receptor
PRISMA: Preferred Reporting Items for Systematic Reviews and Meta-Analyses
PROSPERO: International Prospective Register of Systematic Reviews
PTT: Protein truncation test
RT: Radiotherapy
SBR: Scarff–Bloom–Richardson
SLNB: Sentinel lymph node biopsy
UICC: Union for International Cancer Control
UN: United Nations
UND: Undetermined
