# Supplementary material for: Male breast cancer in Arab populations: a systematic review of molecular, genetic, and clinicopathologic characteristics
Source: J Egypt Natl Canc Inst. 2026 Sep 21;38:75. doi: 10.1186/s43046-026-00408-0 (PMC13594001; doi:10.1186/s43046-026-00408-0)
Supplement: Supplementary file 2 [file 43046_2026_408_MOESM2_ESM.docx]

| **Studies**  **Supplementary Table S3.** **Item-level risk-of-bias assessment of included studies using Joanna Briggs Institute (JBI) critical appraisal checklists.** | **type** | **Q1** | **Q2** | **Q3** | **Q4** | **Q5** | **Q6** | **Q7** | **Q8** | **Q9** | **Q10** | **Q11** | **Risk** |  |
| --- | --- | --- | --- | --- | --- | --- | --- | --- | --- | --- | --- | --- | --- | --- |
| Alsayed et al., 2019 | Case Report | yes | yes | yes | yes | yes | yes | yes | yes |  |  |  | Low |  |
| Hali et al., 2011 | Case Series | yes | unclear | unclear | unclear | yes | yes | yes | unclear | unclear | yes |  | Moderate | |
| Gilbert et al., 2011 | Cohort Studies | yes | yes | yes | unclear | unclear | no | no | yes | yes | yes |  | Moderate |  |
| Elshafiey, 2011 | Cohort Studies | yes | yes | yes | unclear | unclear | yes | yes | yes | yes | yes |  | Low |  |
| El-Habbash, 2009 | Cohort Study | yes | yes | unclear | no | no | yes | yes | unclear | no | yes | yes | Moderate |  |
| Tawil, 2012 | Case Series | yes | yes | yes | yes | yes | yes | No | yes | yes | YES |  | Low |  |
| Guaoua, 2014 | Case Report | yes | yes | yes | yes | yes | yes | NA | YES |  |  |  | Low |  |
| Omari-Alaoui, 2002 | Case Series | yes | yes | yes | unclear | yes | yes | yes | partial | yes | partial |  | Moderate |  |
| Al-saleh, 2011 | Case Report | yes | yes | yes | yes | yes | yes | partial | yes |  |  |  | Low |  |
| Sakhri, 2024 | Case Report | yes | yes | yes | yes | yes | yes | No | yes |  |  |  | Low |  |
| Sekal, 2014 | Case Report | yes | yes | Yes | Yes | Yes | Yes | unclear | Yes |  |  |  | Low |  |
| El Harroudi, 2010 | Case Report | yes | yes | yes | yes | yes | yes | No | yes |  |  |  | Low |  |
| Troudi, 2007 | Cohort Studies | yes | yes | yes | No | No | unclear | yes | NA | NA | NA | yes | Moderate |  |
| Safini, 2016 | Case Report | yes | yes | yes | yes | yes | yes | yes | unclear |  |  |  | Low |  |
| Eshaqi, 2024 | Case Series | No | Yes | Yes | No | No | Yes | Yes | no | unclear | NA |  | Moderate |  |
| Laham, 2022 | Case Report | yes | yes | yes | yes | Yes | Yes | unclear | yes |  |  |  | Low |  |
| Marzogi,, 2025 | Case Report | yes | yes | yes | yes | yes | YES | YES | YES |  |  |  | Low |  |
| Kridis, 2023 | Cohort Studies | unclear | yes | yes | unclear | no | Yes | Yes | Yes | unclear | yes | yes | Moderate |  |
| El Fouhi, 2020 | Cohort Studies | unclear | Yes | yes | unclear | no | Yes | Yes | unclear | unclear | yes | yes | Moderate |  |
| Al-Naqqash, 2025 | Cohort Studies | unclear | yes | yes | unclear | no | Yes | Yes | unclear | unclear | yes | yes | Moderate |  |
| Manai, 2019 | Cohort Studies | unclear | yes | yes | yes | yes | Yes | Yes | unclear | unclear | yes | yes | Moderate |  |
| Abdeljaoued, 2017 | cross-sectional | yes | yes | yes | yes | No | No | yes | yes |  |  |  | Moderate |  |
| Al Awayshih , 2019 | Case Report | yes | yes | yes | yes | yes | unclear | No | yes |  |  |  | Moderate |  |
| Merad, 2024 | cross-sectional | yes | yes | yes | yes | No | No | yes | unclear |  |  |  | Moderate |  |
| El Fouhi, 2022 | cross-sectional | yes | Yes | yes | Yes | No | No | Yes | Yes |  |  |  | Moderate |  |
| Alouani, 2020 | Case Report | Yes | Yes | yes | Yes | Yes | No | NA | Yes |  |  |  | Low |  |
| Laabadi, 2013 | Case Series | Yes | Yes | yes | unclear | Yes | Yes | unclear | No | unclear | Yes |  | Moderate |  |
| Sellal et al., 2011 | Case Series | Yes | yes | yes | unclear | unclear | Yes | Yes | Yes | yes | NA |  | Moderate |  |
| Taha, 2013 | Case Report | yes | yes | yes | yes | unclear | no | no | yes |  |  |  | Low |  |
| Kallel, 2014 | Case Report | Yes | unclear | yes | yes | yes | yes | No | yes |  |  |  | Low |  |
| Sakhri, 2019 | Case Series | unclear | yes | yes | No | No | yes | yes | yes | No | NA |  | Moderate |  |
| Hamdi, 2021 | cross-sectional | yes | yes | yes | yes | yes | unclear | yes | yes |  |  |  | Low |  |
| Awadelkarim, 2007 | cross-sectional | yes | yes | yes | yes | yes | yes | yes | yes |  |  |  | Low |  |
| Kridis-Rejeb, 2020 | Case Series | yes | yes | yes | yes | No | No | yes | yes | No | unclear |  | Moderate |  |
| Al Zouman., 2010 | Case Report | yes | yes | yes | yes | yes | yes | partielly | yes |  |  |  | Low |  |
| Tarrab, 2021 | Case Report | yes | yes | yes | yes | yes | yes | No | yes |  |  |  | Low |  |
| Soliman, 2016 | Cohort Studies | unclear | unclear | yes | yes | No | yes | yes | yes | unclear | unclear | yes | Moderate |  |
| Zebbakh, 2024 | Case Report | yes | unclear | yes | yes | No | No | unclear | yes |  |  |  | Moderate |  |
| Tahrir, 2022 | Case Report | Yes | Yes | yes | Yes | Yes | Yes | Yes | Yes |  |  |  | Low |  |
| El-Beshbeshi, 2012 | Cohort Studies | NA | NA | yes | unclear | No | yes | unclear | yes | yes | yes | yes | Moderate |  |
| Temmim, 2001 | Cohort Study | NA | NA | yes | Yes | Yes | yes | unclear | yes | yes | yes | yes | Low |  |
| Jaheddine, 2024 | Case Report | yes | partial | yes | yes | yes | yes | No | yes |  |  |  | Moderate |  |
| Ekram, 2023 | Case Report | yes | partial | yes | yes | yes | partial | No | yes |  |  |  | Moderate |  |
| Bourhafour, 2011 | Cohort Studies | unclear | NA | yes | yes | yes | yes | yes | yes | unclear | no | yes | Moderate |  |
| Hammedi, 2010 | Case Report | yes | partial | yes | yes | yes | yes | NA | yes |  |  |  | Low |  |
| Alharbi, 2022 | Case Report | yes | yes | yes | yes | yes | yes | NA | yes |  |  |  | Low |  |
| Aldossary, 2019 | Case Report | yes | yes | yes | yes | yes | yes | NA | yes |  |  |  | Low |  |
| Al Salloom et al., 2015 | Case Report | yes | yes | yes | yes | yes | yes | No | Yes |  |  |  | Low |  |
| El-Baradie, 2012 | Cohort Studies | yes | yes | yes | yes | yes | yes | yes | yes | unclear | unclear | yes | Low |  |
| Al-Saleh et al., 2012 | Case Report | yes | unclear | yes | yes | yes | unclear | No | yes |  |  |  | Moderate |  |
| Darouassi et al., 2010 | Case Report | yes | yes | yes | Yes | yes | yes | yes | yes |  |  |  | Low |  |
| Marafi et al., 2020 | Case Report | yes | yes | yes | Yes | NA | yes | NA | yes |  |  |  | Moderate |  |
