# Supplementary material for: Male breast cancer in Arab populations: a systematic review of molecular, genetic, and clinicopathologic characteristics
Source: J Egypt Natl Canc Inst. 2026 Sep 21;38:75. doi: 10.1186/s43046-026-00408-0 (PMC13594001; doi:10.1186/s43046-026-00408-0)
Supplement: Supplementary file 3 [file 43046_2026_408_MOESM3_ESM.docx]

**Table S2** Electronic search strategies used for the systematic literature search across PubMed, Scopus, and Embase.

| **Database** | **Search Strategy** |
| --- | --- |
| **PubMed** | (((("male breast cancer*"[Title/Abstract]) OR ("breast cancer in men*"[Title/Abstract])) OR ("male breast carcinoma*"[Title/Abstract])) OR ("Breast Neoplasms, Male"[MeSH Terms])) NOT (review[Publication Type]) |
| **Scopus** | ( TITLE-ABS-KEY("Male breast cancer*") OR TITLE-ABS-KEY("breast cancer in men*") OR TITLE-ABS-KEY("male breast carcinoma*") OR TITLE-ABS-KEY("breast tumors in men*") ) AND NOT TITLE-ABS-KEY(review) |
| **Embase** | ('male breast cancer*':ab,ti OR 'breast cancer in men*':ab,ti OR 'male breast carcinoma':ab,ti OR 'breast tumors in men*':ab,ti) NOT review:ti,ab,kw NOT review:it |
